# Cognitive-Motor Interference in Multiple Sclerosis: A Systematic Review of Evidence, Correlates, and Consequences

**DOI:** 10.1155/2015/720856

**Published:** 2015-03-09

**Authors:** Douglas A. Wajda, Jacob J. Sosnoff

**Affiliations:** Motor Control Research Laboratory, Department of Kinesiology and Community Health, University of Illinois at Urbana-Champaign, 906 S. Goodwin Avenue, Urbana, IL 61802, USA

## Abstract

Individuals with multiple sclerosis (MS) regularly exhibit deficits in motor and cognitive function. Recent evidence suggests that these impairments are compounded when motor and cognitive task are performed simultaneously such as walking while talking. The changes incurred during simultaneous performance of motor and cognitive tasks are a result of cognitive-motor interference (CMI) and operationalized as dual task costs (DTC). Recently in MS, research has been conducted to understand and analyze the impact of CMI. The purpose of this paper was to review the current literature related to the evidence, correlates, and consequences of CMI in MS. Relevant literature was collected from the results of a PubMed search for terms including “Cognitive-motor interference” or “Cognitive-motor interaction” or “Dual task” and “multiple sclerosis.” Overall, 20 papers were included for review which focused on CMI during balance and walking tasks. The finding that there is a lack of evidence pertaining to changes in the cognitive domain as well as to the specific consequences of CMI in MS was noted. Future work should aim to fill these gaps and ultimately investigate the usefulness of targeted interventions in reducing the deleterious effects of CMI in individuals with MS.

## 1. Introduction

Multiple sclerosis (MS) is a neurodegenerative disease in which focal inflammation causes the degradation of myelin in the nerve fibers of the central nervous system [1]. This damage interrupts the normal transmission of signals along the axons leading to a variety of symptoms [2]. Ultimately, these areas of acute demyelination lead to the degradation and eventual transection of the axons within the CNS [3]. Approximately 400,000 individuals in the U.S. and 2.4 million worldwide are living with MS. Women are 2 to 3 times more likely to be diagnosed with MS and a majority of all cases of MS are confirmed between the ages of 20 to 50 [4].

Among the most common symptoms of MS are motor impairments such as deficits in gait and balance, as well as cognitive dysfunction such as reductions in cognitive processing speed. Approximately 85% of individuals with MS report walking dysfunction to be a major impairment in their daily lives [5]. Balance dysfunction is also regularly reported by individuals with MS even in the absence of clinical disability [6]. Additionally, an estimated 65% of individuals with MS report cognitive deficits [7] which can occur early in the disease process [8].

Previously, motor and cognitive impairments were commonly examined independently of each other. However, research of simultaneous performance of motor and cognitive tasks has identified an interaction between them [9, 10]. Indeed, CMI is common in neurodegenerative disorders and other clinical populations such as dementia [11], stroke [12], Parkinson's disease [13], and MS [14].

It is possible to observe multiple ways in which cognition and motor function interact while performed simultaneously. Predominantly, the changes in performance when cognitive and motor tasks are performed concurrently are termed dual task costs (DTC). These dual task costs represent an operationalization of CMI and are often calculated by computing the percentage change in outcome measures [15] from performance in isolation to dual tasking performance. Plummer and colleagues have outlined nine possible changes observed during the concurrent performance of cognitive and motor tasks [12]. These include four major isolated changes (i.e., motor task facilitation, motor task interference, cognitive task facilitation, and cognitive task interference) as well as the possible combinations of these observations or no changes at all.

Generally, CMI is explained through the use of one of three theoretical frameworks. These include the attentional capacity theory, the bottleneck theory, and a self-awareness theory. Perhaps the most commonly utilized is the attentional capacity theory [10, 16]. This theory maintains an individual has a finite limit on their attentional capacity and given tasks require a certain amount of attentional capacity. If the capacity is reached when multitasking then performance on one or both of the tasks will decline. Similarly, the bottleneck theory suggests that due to limited resources there is a point in information processing where only one task can be performed at a time thus causing decrements when dual tasking [17]. An alternative to these theoretical models is a framework in which self-awareness of limitations and environmental demands elicits a conscious prioritization of one task over the other. Work in other populations has previously shown that older adults tend to prioritize posture (posture first strategy) [18, 19] while dual tasking and in individuals with Parkinson's disease utilize a posture second strategy [20]. To date, no theoretical model for dual tasking has been explicitly tested in individuals with MS. The purpose of this paper was to review the current CMI literature in MS. Particularly, this review focused on the evidence, correlates, and consequences of CMI in this population.

## 2. Methods

The Preferred Reporting Items for Systematic Reviews and Meta-Analysis (PRISMA) statement was used to guide the review [21]. Relevant literature was collected from the results of a PubMed (http://www.ncbi.nlm.nih.gov/pubmed/) search conducted on 09/01/2014. The utilized search terms were “Cognitive-motor interference” or “Cognitive-motor interaction” or “Dual task” and “multiple sclerosis.” Inclusion criteria for the review required studies which were peer reviewed, published in English, assessed CMI in individuals with MS and provided data on both single and dual task performances. Both authors took part in the analysis of the search results and came to a consensus on all articles included in the review. Articles were first screened based on title and abstract and relevant articles thereafter were read and scrutinized in full.

Study quality was assessed through the use of a checklist based on the National Service Framework Typology of Evidence [22]. Each article was rated in relation to five questions developed from this framework: (1) Are the research aims and design clearly stated? (2) Is the study design appropriate for the aims? (3) Are the methods clearly defined? (4) Is the data adequate to support the author's interpretations and conclusions? (5) Are the results generalizable? For each of these questions, a “yes” response was assigned 2 points, an “in part” response was assigned 1 point, and a “no” response assigned 0 points. This provided a 0 to 10 scale on which to categorize each article within the review. Articles receiving ≥7 points were termed high quality, those with 4–6 points were termed medium quality, and articles with ≤3 points were termed low quality.

Data was extracted from each study by a single author (Douglas A. Wajda) and all findings were discussed with the coauthor (Jacob J. Sosnoff). Sample size, MS disability characteristics, motor domain task, cognitive domain task, outcome measures, and general findings were extracted from the selected studies.

## 3. Results

**Figure 1:**
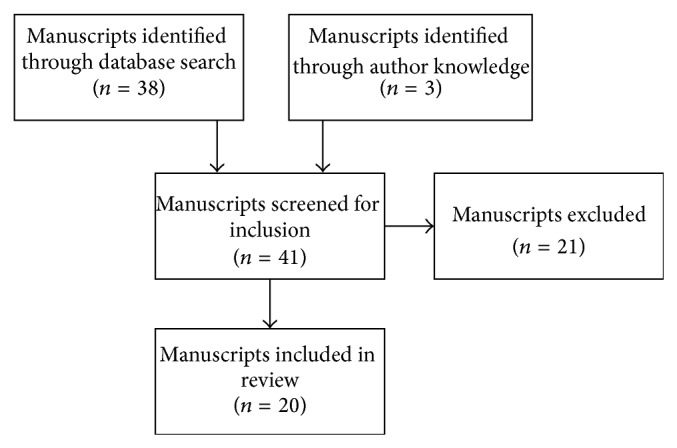
Flow diagram for the determination, screening, and inclusion of relevant articles.

The search returned 38 articles and an additional 3 articles located through the authors' personal knowledge were also included for a total of 41. Both authors took part in the screening of articles based on the stated inclusion criteria. From the original list of 41, 20 articles were included in the current review.  Figure 1 provides a flow chart of this systematic process. Following inclusion, articles were divided into three sections: (1) studies providing evidence of CMI; (2) studies examining correlates of CMI; and (3) studies detailing the consequences of CMI. If applicable, studies could be categorized into multiple categories. For reference throughout the current article, DTCs were calculated using the percentage change from single to dual task conditions for those studies that did not explicitly report DTC values. Additionally, for those studies utilizing multiple cognitive tasks an average DTC is presented.

The articles included in the review all displayed medium to high quality based on the predefined checklist. Quality ratings for each study are presented in Tables 1 and 2. Overall, 80% of the articles were rated as high quality with the other 20% being medium quality. Based on the recommendations from the National Service Framework [22], the final group of papers received a research grade of A indicating that high quality articles with direct applicability to the systematic review were utilized.

The majority of studies investigating CMI in MS have been cross-sectional and examined changes in walking and/or balance performance with the addition of a cognitive task. The analysis of CMI during other motor tasks such as isometric finger contractions has also been completed [23]. Generally, the analysis of performance on the cognitive task represented a secondary outcome or was not determined at all. A large variety of cognitive tasks with specific cognitive loads were used to evaluate CMI across the studies.

Walking represents the most commonly tested motor task during CMI investigations in MS [24–37].  Table 1 outlines the characteristics (sample size, sample disability status, and utilized cognitive and motor tasks) for the identified studies using gait tasks. Seventy percent of the studies included in the current review utilized walking velocity as the main outcome measuring for detecting changes in motor performance.

Balance tasks represented another common experimental approach for the evaluation of CMI in individuals with MS. While fewer studies (*n* = 7) have examined the interrelationship between postural control and cognition compared to walking tasks, there is evidence of CMI in balance tasks in MS [24, 38–43]. Details for the studies which utilized balance measures as the primary motor task are presented in Table 2.

## 4. Discussion

### 4.1. Evidence of CMI during Walking Tasks

**Figure 2:**
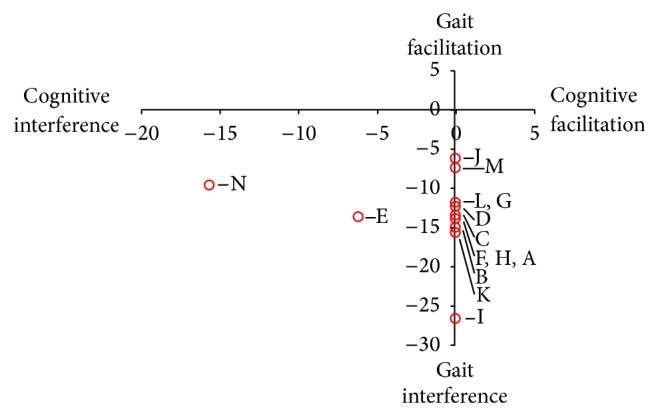
Graphical representation of the DTCs for the included studies utilizing walking as the main motor outcome.

Figure 2 depicts the collective DTCs from the 14 investigations examining CMI during walking in MS with over 750 participants across the disability spectrum. Each investigation with a reported finding of CMI in walking is indicated with an open circle (studies with overlapping results are represented with a single point). For each study, the abscissa represents change in cognitive task performance while the ordinate represents changes in gait from single to dual task conditions. It is clear in the figure that the main observation across this body of work is that the primary effect of CMI during walking in individuals with MS is motor interference (i.e., decrease in gait velocity). Percentage declines in gait speed with the simultaneous performance of cognitive task ranged from ~6% to ~27%. Additionally, Figure 2 further highlights the lack of information presented in regards to performance in the cognitive domain. The following summaries of findings were selected to offer a general scope of CMI during walking in MS across varying disability levels and task conditions.

Hamilton and colleagues reported one of the first walking while talking papers in MS in 2009 [37]. The cross-sectional analysis showed that individuals with MS had slowed gait velocity and diminished cognitive task performance compared to controls when performing both a titrated and fixed length digit span task. Interestingly, the study also revealed an increase of swing time variability in individuals with MS from single to dual task conditions. Gait variability during nondistracted walking has shown to be higher in recurrent MS fallers than in nonfallers [44]. However, the connection between gait variability during dual task conditions and falls in individuals with MS has not been explicitly examined.

Following the results of Hamilton et al., a multitude of studies examining dual task costs in MS have been conducted. These studies have been carried out generally during short walking tasks (~10 m); however, various cognitive tasks have been utilized. Word list generation represents one of the most prominent tasks employed [26–31, 35, 36] as well as subtraction and counting tasks [28, 29, 32–34].

In addition to varied testing methodologies, participants with a wide range of disability levels as indexed by scores on the Expanded Disability Status Scale (EDSS) [45] have also been observed in these studies. For instance, Kalron et al. investigated CMI in individuals with clinically isolated syndrome (CIS) indicative of MS with an average expanded disability status scale of 1.7 [36]. Overall, it was found that the CIS group had greater DTC during walking than healthy age matched controls. Contradictory to this finding, Allali et al. determined that there was no significant difference in individuals with MS who had low disability levels (EDSS mean ± standard deviation = 1.9 ± 1.0) and healthy controls [28]. In one of the first studies to include a wide range of disability, Sosnoff and colleagues observed CMI in MS in individuals with mild (EDSS 2.0–3.5), moderate (EDSS 4.0–5.5), and severe (EDSS 6.0–6.5) disability [35]. The primary finding of this report was significantly greater DTCs of walking in the severe and moderate disability group compared to the mild disability group.

Somewhat surprisingly, few studies computed the effect of dual tasking on performance of the concurrent cognitive task. To date only 2 out of 14 investigations in CMI in MS during walking have quantified single task performance of the cognitive task allowing for the calculation of DTCs of cognition [28, 37]. Both studies on average observed diminished performance of the cognitive task during dual task conditions ranging from ~6% to ~16% [37]. Moreover, preliminary work from our group has found that the DTCs of cognition (response accuracy and utterances) during walking with while performing an alternating letter task show moderate and large negative correlations respectively with the Activities Specific Balance Confidence (ABC) Scale [46]. The results suggest that those individuals with low self-perceived balance confidence exhibit greater decreases on the cognitive task during dual task conditions than those with higher confidence.

### 4.2. Evidence of CMI during Balance Tasks

**Figure 3:**
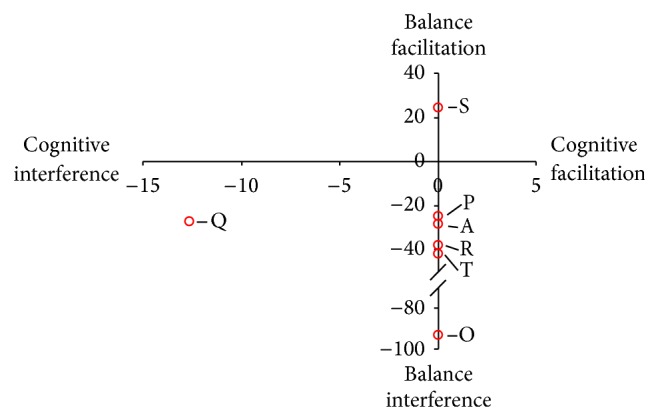
Graphical representation of the DTCs for the include studies utilizing balance tasks as the main motor outcome.

Figure 3 provides an overview of the observed changes from the cited DTC of balance studies identified in Table 2. Similar to the walking studies, there is little evidence regarding the changes to cognitive performance during dual task balance testing. One study identified a decrease in Stroop reaction time and accuracy when performed concurrently with a dynamic stepping task [40].

The majority of CMI investigations utilizing balance tasks in MS have taken place in individuals with low levels of disability [39, 42, 43]. In general, the primary outcome measures of these studies have been center of pressure (COP) metrics (e.g., sway area and sway rate) generated during standing balance trials completed on a force platform [24, 38, 39, 41–43]. Additionally, dynamic balance tasks such as stepping have also been investigated [40]. The following articles were chosen to highlight results of CMI during balance tasks in MS based on disability status and outcome measure selection.

Kalron et al. determined that an added cognitive task resulted in increased sway velocity in individuals with CIS, thus providing further evidence that CMI is present even in the earliest stages of the disease process [43]. Following these earlier findings, Boes et al. aimed to investigate the impact of disability levels on CMI during standing balance [41]. Utilizing force platform metrics they determined that while individuals with higher disability levels had worse postural control than those with low disability, there was no distinct effect of a simultaneous cognitive task on these relationships. That is, calculated DTCs were not greater in individuals with higher disability during balance tasks compared to the mild disability group.

Multiple studies have gone beyond the analysis of changes in standard force platform COP measures to evaluate nonlinear metrics and dynamic balance tasks. In a series of studies, Negahban and colleagues have observed that the addition of cognitive tasks during balance cause the COP to become less regular and more complex [39] while also resulting in a decrease of variability in COP velocity suggesting a lack of flexibility when adapting to postural perturbations [42]. Finally, Jacobs and Kasser reported on the impact of CMI during dynamic balance tasks [40]. Namely, stepping response was hindered in individuals with MS during dual task due to delayed anticipatory postural adjustment onsets.

### 4.3. Correlates of CMI

While a great deal of research regarding CMI in MS has focused on the observation of the phenomena itself under various testing conditions, there is ongoing research investigating the possible factors related to dual task changes. The motivation of this body of work is to inform future rehabilitation strategies. These analyses have looked primarily at correlations between DTC and measures of disability, mobility, cognition, and MS symptoms. These investigations have produced mixed results.

Disability status represents a frequently utilized measure in exploratory analysis focusing on factors related to DTC. Commonly in MS research, disability is indexed with the neurologist administered expanded disability status scale (EDSS) [45] as well as self-reports of disability (SR-EDSS). Previous reports have observed that higher disability levels were associated with larger DTCs of walking velocity [26, 35]. In contrast, another recent investigation with similar range of disability scores (median EDSS = 4, IQR = 2.75) found no correlation between EDSS scores and DTCs of spatiotemporal gait parameters [25]. Furthermore, Hamilton also found no relationship between DTC and disability as measured by the EDSS [37] although the sample only contained individuals who did not require an assistive device for walking (EDSS Range = 0.0–5.5). A possible reason for the inconsistent findings could be related to the methodological differences between investigations including differences in quantification of disability (e.g., self-report versus clinically determined) and cognitive task utilized.

Symptom and demographic factors such as fatigue, depression, spasticity, pain, age, education, and disease duration have also been examined as correlates of DTC of walking. One investigation [37] observed a relationship between dual task cost in walking with fatigue. Conversely, the reports of Learmonth et al. and Motl et al. did not observe any correlations between DTCs and symptoms including fatigue [26] or demographics [25].

In addition to disability, symptoms, and demographic characteristics, mobility and cognition have been examined as correlates of CMI of walking. Walking tests in MS generally consist of tests of walking speed such as the timed twenty-five foot walk and walking endurance as quantified by the six minute walk. Indeed, performance on both these measures has been shown to be correlated with DTCs of gait [26, 27] suggesting that general mobility performance influences the impact on walking when adding a concurrent cognitive challenge. Similarly, cognitive processing speed as determined by the symbol digit modalities test has also been shown to correlate with the DTC of walking velocity [26].

Correlates of DTCs of balance have received much less scrutiny. To date, one study has examined the correlates of DTCs of standing balance in MS [38] and a second examined the impact of fatigue on dual tasking during a cued stepping task requiring participants to take a step with their preferred leg followed by one with the opposite leg upon seeing a visual “GO” stimuli [40]. Wajda et al. measured postural sway during static balance tests on a force platform in 62 individuals with MS. Participants performed standing balance assessment in isolation and in dual task trials with an added word list generation task. Participants also completed the Berg Balance Test [47] and the ABC Scale. The researchers observed no significant correlations between DTC of standing balance and symptoms or the clinical balance measures [38]. Moreover, the authors observed an apparent ceiling effect in sway area with only those individuals who had low baseline sway actually increasing their sway area during dual task conditions. It was proposed that this was due to the individuals with diminished balance being unable to increase sway without exceeding their limits of stability and indirectly suggests a posture first strategy. In another investigation of 13 individuals with MS a significant correlation between patient reported fatigue (MFIS) and dual task related increases in anticipatory postural adjustment onset times and foot-lift onset was observed [40].

### 4.4. Consequences of CMI

Despite the growing evidence suggesting that both motor and cognitive deficits are compounded by CMI in MS, there is little evidence of the direct consequences of these changes. CMI has previously been suggested to be related to falls in other clinical populations including older adults [48] and Parkinson's [49]. In MS, the relationship between falls and CMI is not fully clear. One cross-sectional analysis consisting of 33 individuals with MS with a history of falls determined that higher DTCs of walking were indicative of a greater risk for future falls based on a physiological fall screening test [30]. A second study with over 150 participants found that changes in walking speed during a ten meter walk task while computing subtractions of sevens was not predictive of future falls in MS [32]. It is of note, however, that another observational study consisting of 76 individuals with MS reported that the performance of a timed up and go test with added cognitive challenge was predictive of future accidental falls [50]. While single task performance of the TUG was not included to calculate DTC for the current review, the results suggest a possible relationship between cognitive-motor interference and falls in MS. Finally, the discrepancies between these findings in relation to falls could be a result of the varied testing procedures based on both task (timed up and go versus walking) and walking speed (normal versus fastest) as well as cognitive task. It is also important to note that there is no extant data on cognitive DTCs and consequences in persons with MS. Given the association between cognition and falls in MS [51, 52], this is an important topic for future inquiry.

Furthermore, tasks that involve both motor and cognitive resources represent a large portion of day to day activities. These can include, for example, carrying on a conversation while walking with a friend or crossing a busy street. The ability to easily complete these tasks may be further hindered by CMI in multiple sclerosis. To date, only one study has directly investigated the effectiveness of a targeted intervention on CMI in MS. The pilot study (*n* = 9) examined the effect of one year of treatment with natalizumab on the dual task related changes of gait in MS [29]. The primary observation of this work was a reduction in DTCs of gait velocity, stride length, and stride time following the treatment. Additionally, it is of note that these changes occurred without a corresponding change in participants' single task walking velocity suggesting an improvement to dual tasking ability not solely mobility. The findings may also indicate that DTCs are more sensitive to slight physiological or disability status changes than gait measurement in isolation. In light of these encouraging changes of DTC with disease modifying treatment, it still remains to be seen if a targeted dual task walking and balance intervention would also reduce CMI in MS. Evidence from other populations suggests that the regimented practice of dual tasking can indeed decrease CMI [53].

## 5. Conclusions

The purpose of this paper was to review the current CMI literature in MS. Particularly, this review focused on the evidence, correlates, and consequences of CMI in this population. Despite the broad range in MS symptoms and severity, CMI appears to occur throughout the population. In general, CMI serves to compound the already existing impairments in MS such as walking, balance, and cognition. While a large amount of work has gone into the observation of the phenomena, more room is available for studies that seek to examine the correlates and primarily the consequences of CMI in individuals with MS.

It is proposed that future work regarding CMI in individuals with MS seeks to fill the stated gaps in the current literature. Specifically, there remains a need to directly test the theoretical frameworks associated with CMI as those results could ultimately inform the development of interventions aimed at reducing the compounding effects of dual tasking in this population. Additionally, further investigation is warranted towards the understanding of the impact of dual tasking on the cognitive domain in MS. Moreover, further analysis is necessary regarding the direct consequences of CMI in MS such as its relationship to falls and fall risk. As most everyday activities including some aspect of cognitive-motor dual tasking, it is imperative to further understand CMI in MS and ultimately determine if adaptations can be made to current clinical practice to help reduce its effects.

## Figures and Tables

**Table 1:** Characteristics of studies utilizing walking tasks during dual tasking.

Study ID	Author	Publication year	Sample size	EDSS	Motor outcome	Cognitive task	Quality
A	Kramer et al. [24]	2014	61	3.0 ± 1.0^a^	Walking velocity	Questions (How many sides on a cube?)	High
B	Learmonth et al. [25]	2014	61	4.0 (2.8)^b^	Walking velocity	Alternating letters (A, C, E)	High
C	Motl et al. [26]	2014	82	3.5 (3.0)^b,d^	Walking velocity	Word List Generation	High
D	Sosnoff et al. [27]	2014	96	4.5 (3.0)^b^	Walking velocity	Word list generation	High
E	Allali et al. [28]	2014	25	1.9 ± 1.0^a^	Walking velocity	Word list generation/counting	High
F	Allali et al. [29]	2014	9	2.9 ± 1.1^a^	Walking velocity	Word list generation/counting	Medium
G	Wajda et al. [30]	2013	33	6.0 (2.0)^b,d^	Walking velocity	Word list generation	High
H	Wajda et al. [31]	2013	10	2.5–4.0^c^	Walking velocity	Word list generation	Medium
I	Gunn et al. [32]	2013	148	3.5–6.5^c^	Walking velocity	Serial 7's	High
J	Nogueria et al. [33]	2013	12	0.0–1.5^c^	Walking velocity	Serial 3's	Medium
K	Nogueria et al. [34]	2013	120	2.7 ± 2.0^a^	Walking velocity	Serial 3's	High
L	Sosnoff et al. [35]	2011	77	2.0–6.5^c^	Walking velocity	Word list generation	High
M	Kalron et al. [36]	2010	52	1.7 ± 0.2^a^	Walking velocity	Word list generation	High
N	Hamilton et al. [37]	2009	18	2.7 ± 1.6^a^	Walking velocity	Fixed and titrated digit span recall	High

Notes: ^a^Mean ± SD; ^b^median (IQR); ^c^range; ^d^self-reported.

**Table 2:** Characteristics of studies utilizing balance tasks during dual tasking.

Study ID	Author	Publication year	Sample size	EDSS	Motor outcome	Cognitive task	Quality
O	Wajda et al. [38]	2014	62	6.0 (2.0)^b,d^	COP sway area	Word list generation	High
A	Kramer et al. [24]	2014	61	3.0 ± 1.0^a^	Center of force displacement (single leg)	Random number typing	High
P	Negahban et al. [39]	2013	23	2.5 ± 1.1^a^	Recurrence quantification analysis	Silent serial 3's with endpoint recall	Medium
Q	Jacobs and Kasser [40]	2012	13	0–4.5^c^	Step initiation time	Auditory stroop task	High
R	Boes et al. [41]	2012	45	2–6.5^c^	COP sway area	Word list generation	High
S	Negahban et al. [42]	2011	23	2.5 ± 1.1^a^	COP sway area	Silent serial 3's with endpoint recall	High
T	Kalron et al. [43]	2011	52	1.7 ± 0.2^a^	COP sway rate	Visual stroop task	High

Notes: ^a^Mean ± SD; ^b^median (IQR); ^c^range; ^d^self-reported.
